# A wide posterior release is associated with better kyphosis restoration in the surgical treatment of adolescent idiopathic scoliosis

**DOI:** 10.1007/s43390-025-01143-7

**Published:** 2025-07-10

**Authors:** S. Ohrt-Nissen, M. Heegaard, L. Ragborg, N. Tøndevold, T. B. Andersen, M. Gehrchen, B. Dahl

**Affiliations:** Spine Unit, Department of Orthopedic Surgery, Rigshospitalet, Copenhagen University Hospital, Copenhagen, Denmark

**Keywords:** Adolescent idiopathic scoliosis, Surgery, Thoracic kyphosis, Kyphosis restoration, Release

## Abstract

**Purpose:**

To examine whether the use of a wide posterior osseo-ligamentous release in adolescent idiopathic scoliosis (AIS) improves restoration of thoracic kyphosis.

**Methods:**

We retrospectively included a consecutive cohort of AIS patients undergoing surgical treatment involving the thoracic spine (Lenke 5 excluded) over two consecutive time periods. The first time period served as control group. In the second time period, standard surgical technique was supplemented with a wide posterior release of the lamina, spinous process and supraspinous ligaments (no removal of the inferior facet) at 4–5 levels corresponding to the apex of the thoracic curve. Patients were categorized as preoperatively hypo- or normokyphotic and intraoperative data, and 2-year postoperative radiographic data were recorded.

**Results:**

We included 191 patients. Mean age was 15.8 ± 2.3 years, and mean Cobb angle was 60 ± 12°. Sixty-two (32%) patients were classified as hypokyphotic (global kyphosis ≤ 30°) preoperatively. Baseline coronal and sagittal parameters were similar between the posterior release and control group. In the hypokyphotic group, the use of posterior release resulted in an increase in kyphosis from 19 ± 7° to 38 ± 11° vs. 22 ± 8° to 32 ± 7° in the control group (*p* = 0.018). 15% vs. 49% was hypokyphotic at 2-year follow-up (*p* = 0.020). In the preoperatively normokyphotic group, the change in kyphosis was 6 ± 9° vs − 1 ± 10° (*p* < 0.001) in the posterior release and control group, respectively, but with no difference in the final 2-year kyphosis (47 ± 8° vs. 46 ± 10°). Two-year major coronal Cobb angle was 28 ± 9° vs. 26 ± 9° in the posterior release and control group, respectively (*p* = 0.206). Median intraoperative blood loss was 500 (IQR: 412–743) ml vs. 600 (IQR: 500–900). There was one case of neurological injury in the control group and none in the posterior release group.

**Conclusion:**

The use of a posterior osseo-ligamentous release results in an increased thoracic kyphosis restoration in preoperatively hypokyphotic patients without increasing blood loss or the risk of neurological injury.

## Introduction

Adolescent idiopathic scoliosis (AIS) is characterized by a coronal scoliosis, axial rotation and varying degrees of hypokyphosis. The fundamental goal of corrective surgery for spinal deformity is to achieve a balanced spine requiring a minimal amount of energy expenditure [1]. Current gold standard of surgical treatment of AIS is all-pedicle screw constructs providing stable correction both in the coronal and axial plane. However, the substantial derotation results in a lengthening of the anterior column as well as an inevitable flattening of the concave rod. This increases the risk of failure to restore kyphosis or even causing iatrogenic hypokyphosis in otherwise normokyphotic patients [2–4].

Biomechanical studies have shown that resection of all three ligaments of the posterior ligamentous complex provides a substantial release resulting in an increased range of motion of over 25% in passive flexion (kyphosis) [5]. Theoretically, kyphosis restoration could be increased by a more extensive posterior release such as multiple Ponte osteotomies excising the lamina, superior- and inferior facet. Faldini et al. performed a systematic review on the effect of Ponte osteotomies and concluded that they allow for significant restoration of kyphosis in hypokyphotic AIS curves, but that it may come at the expense of significantly greater blood loss and a higher complications rate [6]. Ponte osteotomy is by nature a closing osteotomy but the goal of release in AIS is to lengthen the posterior column, which would require an opening osteotomy. We hypothesized that a wide posterior osseous release, without removing the inferior facet, would achieve the goal of kyphosis restoration without increasing blood loss or risking neurological injury.

It has become increasingly clear that the sagittal plane is essential to address during surgery in pediatric deformities [7, 8]. Achieving optimal sagittal alignment should be a key objective of surgery, as postoperative hypokyphosis is unsatisfactory to the patient and may affect the patient’s long-term quality of life [9, 10]. The aim of the current study was to assess the effect of a wide posterior release on postoperative sagittal alignment after surgical treatment of AIS.

## Methods

**Fig. 1:**
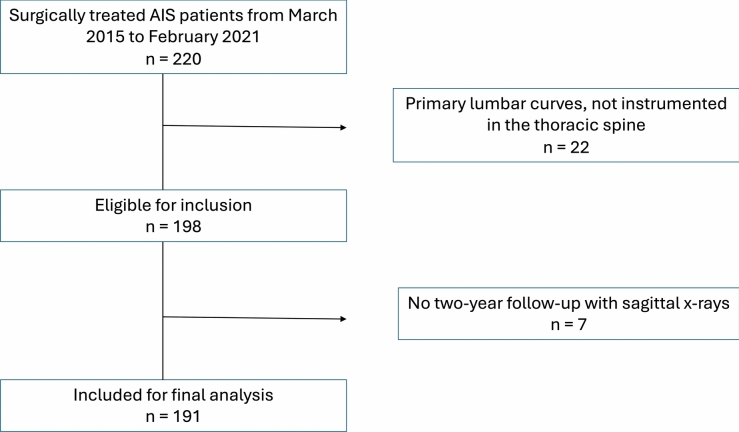
CONSORT flow chart showing the inclusion process

The study was approved by the National Patient Safety Authority (case ID: 31-1521-327). We retrospectively included a consecutive cohort of AIS patients undergoing surgical treatment involving the thoracic spine (Lenke 5 excluded) over two consecutive time periods (Fig. 1). This was a single-center study. All surgeries were dual-surgeon (two of six different surgeons). All patients were treated using the same standardized surgical technique: partial facetectomies with complete removal of the inferior facet were performed on all intended fusion levels. The superior facet was not removed. Segmental uniplanar low-profile pedicle screws were used for fixation. Differential rod contouring with rod derotation and direct vertebral rotation was performed. The convex rod was bend to the target thoracic kyphosis. We used a high stiffness construct aiming for a screw density of 2.0 and in all cases. We used beam-like cobalt-chromium rods with a transition to a standard circular rod at the three cranial instrumented levels.

**Fig. 2:**
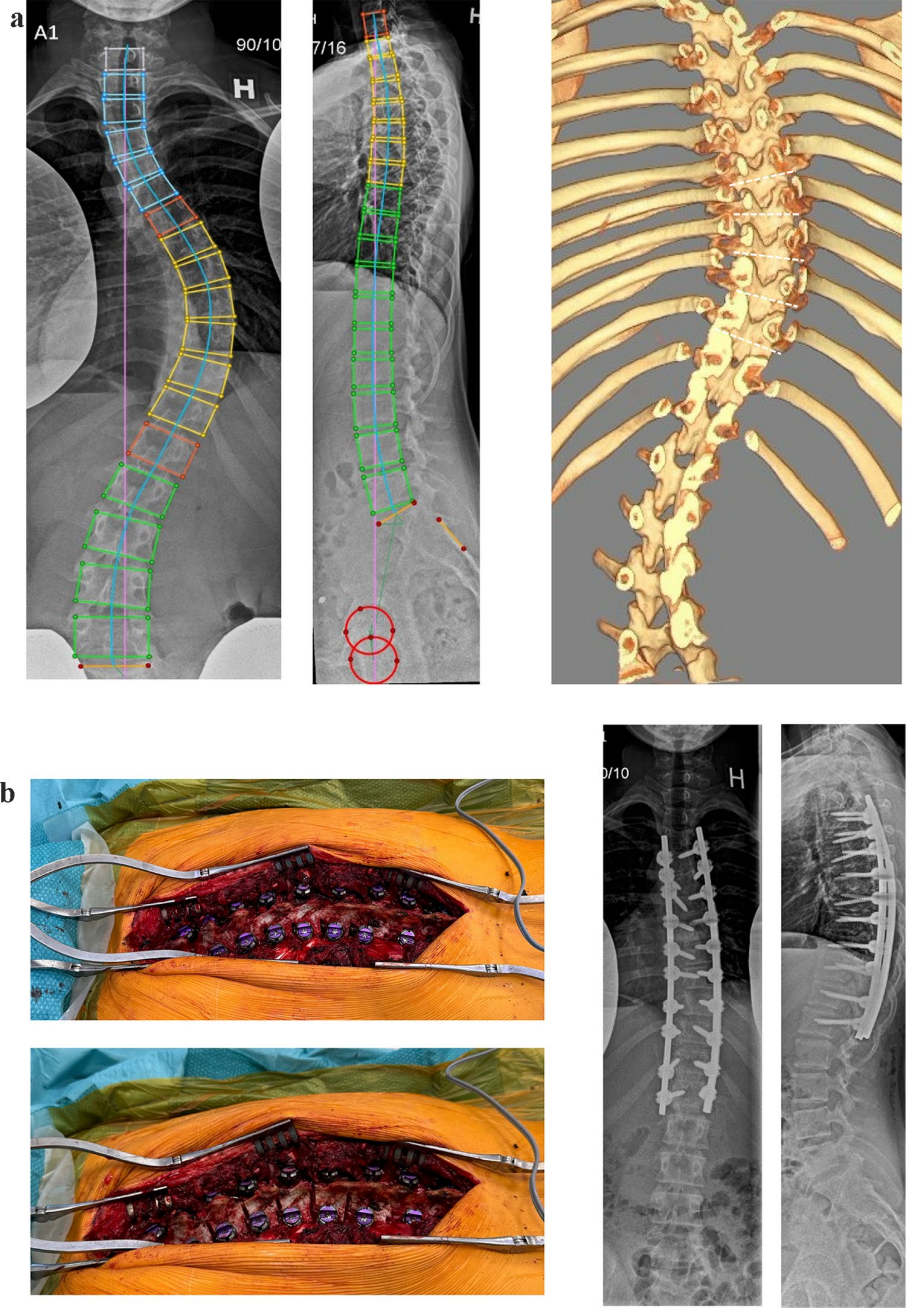
**a** Preoperative X-ray of a Lenke type 1A curve with a substantial hypokyphosis. On the right is the 3-D reconstructive CT suggesting the site and trajectory of the posterior release corresponding to the apex of the curve (dotted lines). **b** Intraoperative image of the thoracic spine before (left) and after (right) posterior release. Postoperative X-ray showing normalization of thoracic kyphosis

The first time period (March 2015 to May 2018) served as control group. In the second time period (June 2018 to February 2021), the standard surgical technique was supplemented with a wide posterior release of the lamina, spinous process and supraspinous ligaments (no removal of the inferior facet) at 4–5 levels corresponding to the apex of the thoracic curve (Fig. 2).

All images were uploaded to the same radiographic software (KEOPS, SMAIO) [11], and radiographic measurements were done by a single investigator (S.O.) with more than a decade of experience in diagnosis and treatment of pediatric deformities.

We categorized patients based on the preoperative sagittal X-ray as either hypokyphotic or normokyphotic based on a 30-degree threshold of global kyphosis, which was two standard deviations below the mean value of skeletally mature young adults [12].

### Statistical analysis

Data analysis was performed in R version 4.0.3. Categorical data are presented by frequencies and related percentages. Continuous data are reported as a means with standard deviation. Continuous data were analyzed using unpaired t test or Wilcoxon rank sum test and categorical variables were compared using Pearson Chi-squared test. The significance level was set at 0.05.

## Results

We included 191 patients, 103 from the first period, 88 patients from the second period. Mean age was 15.8 ± 2.3 years, and mean major Cobb angle was 60 ± 12°. 87% of patients were female, and mean BMI was 20.3 ± 3.2 with no differences between the groups (p > 0.653). Sixty-five percent were Lenke type 1, 15% type 2, 9% type 3 and 11% type 6.

**Fig. 3:**
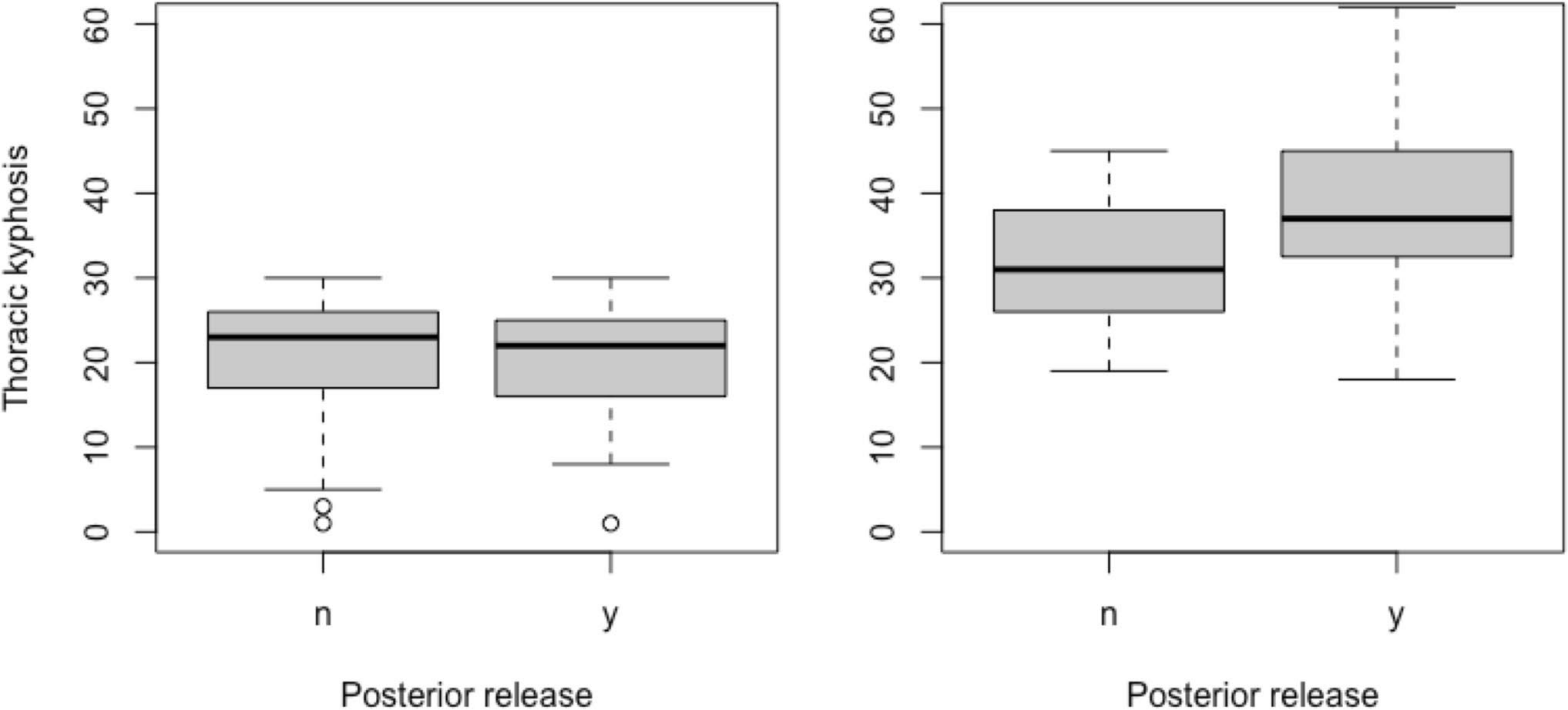
Preoperative (left) and 2-year postoperative (right) thoracic kyphosis with and without posterior release. From the preoperatively hypokyphotic group

Preoperatively, 62 patients were classified as hypokyphotic (global kyphosis ≤ 30°) (Table 1). In the hypokyphotic group, the use of posterior release resulted in an increase in kyphosis from 19 ± 7° to 38 ± 11° vs. 22 ± 8° to 32 ± 7° in the control group (*p* = 0.018). 17% vs 50% were hypokyphotic at 2-year follow-up (= 0.020) (Fig. 3). The absolute risk reduction when using posterior release was 33.8% (95%12.5–55.1) corresponding to a numbers-needed-to-treat of 3 (95% CI: 2–9) to avoid one case of postoperative hypokyphosis.

**Table 1:** Preoperatively hypokyphotic group

	No posterior release *n* = 35	Posterior release *n* = 27	*p*-value
*Preoperative*			
Age, years	16.0 ± 2.0	15.4 ± 2.5	0.356
Major curve, °	59 ± 12	58 ± 11	0.699
Major curve flexibility, %	43 ± 14	45 ± 13	0.737
Global balance, mm	15 ± 13	16 ± 13	0.859
Secondary curve, °	39 ± 9	40 ± 11	0.874
Pelvic incidence, °	53 ± 12	50 ± 9	0.316
Pelvic tilt, °	9 ± 10	8 ± 6	0.549
Sacral slope, °	44 ± 7	43 ± 8	0.457
Thoracic kyphosis, °	22 ± 8	19 ± 7	0.578
Lumbar lordosis, °	60 ± 9	57 ± 9	0.258
Sagittal vertical axis, mm^1^	− 20 (29)	− 12 (34)	0.376
*Two-year follow-up*			
Major curve, °	25 ± 7	31 ± 10	0.007
Global balance, mm	10 ± 9	12 ± 10	0.409
Secondary curve, °	18 ± 8	22 ± 9	0.095
Pelvic incidence, °	52 ± 11	51 ± 9	0.859
Pelvic tilt, °	9 ± 9	8 ± 8	0.949
Sacral slope, °	43 ± 7	42 ± 8	0.542
Thoracic kyphosis, °	32 ± 7	38 ± 10	0.003
Change in kyphosis from preop. to 2-year follow-up	10 ± 8	18 ± 9	0.001
Lumbar lordosis, °	62 ± 10	64 ± 10	0.396
Hypokyphotic ± < 30°, no	17 (49%)	4 (15%)	0.020
Sagittal vertical axis, mm^1^	− 11 (34)	− 7 (33)	0.668

Data reported as mean ± SD unless otherwise specified

^**1**^Negative values mean the plumb line falls behind the S1 endplate

In the preoperatively normokyphotic group, the 2-year kyphosis was 47 ± 8° vs. 46 ± 10° in the posterior release and control group, respectively (*p* = 0.601) (Table 2).

**Table 2:** Preoperatively normokyphotic group

	No posterior release *n* = 68	Posterior release *n* = 61	*p*-value
*Preoperative*			
Age, years	16.4 ± 3.4	16.0 ± 1.8	0.382
Major curve, °	61 ± 13	58 ± 12	0.125
Major curve flexibility, %	42 ± 20	46 ± 22	0.409
Global balance, mm	15 ± 14	19 ± 12	0.147
Secondary curve, °	42 ± 12	39 ± 10	0.137
Pelvic incidence, °	46 ± 14	45 ± 10	0.554
Pelvic tilt, °	6 ± 7	7 ± 7	0.442
Sacral slope, °	40 ± 11	38 ± 8	0.178
Thoracic kyphosis, °	47 ± 11	42 ± 8	0.005
Lumbar lordosis, °	65 ± 14	62 ± 11	0.145
*Two-year follow-up*			
Major curve, °	26 ± 10	27 ± 9	0.829
Global balance, mm	11 ± 8	15 ± 12	0.065
Secondary curve, °	21 ± 10	20 ± 10	0.774
Pelvic incidence, °	46 ± 13	46 ± 10	0.708
Pelvic tilt, °	7 ± 8	7 ± 7	0.752
Sacral slope, °	39 ± 12	39 ± 9	0.510
Thoracic kyphosis, °	46 ± 10	47 ± 8	0.601
Change in kyphosis from preop. to 2-year follow-up	− 1 ± 10	6 ± 9	< 0.001
Lumbar lordosis, °	61 ± 14	63 ± 10	0.647
Hypokyphotic ± < 30°, no	4 (6%)	2 (3%)	0.740

Data reported as mean ± SD unless otherwise specified

Two-year major coronal Cobb angle was 28 ± 9° in the posterior release group vs. 26 ± 9° in the control group (p = 0.206). There were no differences in coronal balance or secondary curve size.

Median intraoperative blood loss was 500 (IQR: 412–743) cc vs. 600 (IQR: 500–900) cc in the posterior release and control group, respectively (*p* = 0.838). The percentage of estimated blood volume lost was 20 ± 9% compared to 17 ± 6% (*p* = 0.216). There was one case of neurological injury in the control group and none in the posterior release group.

## Discussion

The results of this study demonstrate that a wide posterior osseo-ligamentous release can significantly improve kyphosis restoration in surgical treatment of AIS, particularly for patients with a substantial preoperative hypokyphosis. The reduction in the proportion of patients remaining hypokyphotic at 2-year follow-up (15% vs 49%) further underscores the efficacy of this technique. This improvement likely stems from the increased flexibility afforded by the wide posterior release, allowing for better rod contouring and derotation without sacrificing sagittal alignment [13]. Pizones et al. examined 80 Lenke type 1 AIS patients and found, similar to our study, no effect of Ponte osteotomies in the normokyphotic group. In the hypokyphotic group, though, Ponte osteotomies resulted in a kyphosis increase of 11° [14]. Wang et al. and Feng et al. found similar effect on kyphosis restoration but showed an increased blood loss of 120 cc and a longer operating time in the Ponte osteotomy group [15, 16].

Our findings have potentially important implications for surgical technique and patient outcomes in AIS treatment. An increase of almost 20 degrees represents a substantial improvement although the clinical implication of normalizing kyphosis is not well established in the adolescent population. It is important to note that the no-release group also showed a mean improvement in kyphosis (10 degrees). The difference in kyphosis restoration was 8 degrees between the two groups (Table 1).

Several studies have shown that a failure to restore kyphosis results in a reciprocal loss of lordosis at follow-up, which may have a detrimental effect on the long-term outcome [17, 18]. Postsurgical hypokyphosis may increase the risk of distal junctional kyphosis and proximal junctional kyphosis as well as positive sagittal balance and higher rates of low back pain [19, 20]. Young et al. found that at 30-year follow-up, patients with a thoracic hypokyphosis had a substantially higher rate of cervical disc degeneration in adulthood [21]. Bernstein found a higher degree of lumbar disc degeneration in AIS patients with a thoracic flat back after surgery [22].

In the preoperatively normokyphotic patients, we saw the small kyphosis increase after posterior release with no change in the control group. However, the final kyphosis was similar between the groups (47° vs 46°), and few patients were hypokyphotic at follow-up (3% vs. 6%). This suggests that the technique primarily benefits those with preexisting hypokyphosis, while not overcorrecting those with normal kyphosis.

The comparable 2-year major coronal Cobb angles (28° vs 26°) indicate that the posterior release technique does not in itself compromise coronal plane correction. This is crucial, as maintaining sufficient coronal correction while improving sagittal alignment has been a challenge in AIS surgery [23]. A higher coronal major curve was seen in the posterior release group at 2-year follow-up in the hypokyphotic group (31° vs 25°). Due to increased focus on achieving a normalized kyphosis, coronal segmental distraction or similar methods were used rarely to avoid flattening out the spine in the sagittal plane. Theoretically, a release of the posterior ligamentous complex could result in an increased apical deviation toward the convexity of the curve. Focus should be on maintaining a sufficient coronal correction when employing the posterior release technique.

The long-term clinical significance of postoperative hypokyphosis remains unclear. Johnson et al. identified a correlation between hypokyphosis and reduced pulmonary function [24]. In a study by Matsumoto et al. involving 123 patients who underwent selective fusion with a 2-year follow-up, 31% experienced postoperative loss of kyphosis, which was strongly associated with a low degree of lordosis [17]. Definitions of hypokyphosis and methods for measuring kyphosis vary widely in the literature. The Lenke classification uses T5–T12 as a standard segment for kyphosis measurement [25], but Roussouly and colleagues have shown in multiple studies that spinal shape and extent of kyphosis differ greatly among individuals, suggesting a fixed parameter to be of limited value [26]. Several publications have established individualized ‘global kyphosis’ values based on normative data [27], but a pathological threshold in the adolescent and young adult population has not been established. Since spinal fusion often extends beyond T5–T12, we determined that global kyphosis was the most appropriate measure. In the absence of a defined pathological threshold for hypokyphosis in AIS, we chose 2 standard deviations below the mean in the background population as an expression of failure to correct the kyphosis to within a normative range.

A multicenter study from the Harms study group reported that 80% of AIS patients were hypokyphotic following posterior instrumentation [28]. When evaluating factors influencing kyphosis restoration, the study found no association with rod type, screw density, or preoperative kyphosis. The only significant predictor was the individual surgeon, highlighting the critical importance of surgical technique. The authors observed that the most effective surgeons consistently used Ponte osteotomies, though this association did not reach statistical significance. Our study did not assess external validity, and further research is needed to determine whether our findings are consistent across other surgeons and patient groups.

### Safety considerations

A key finding is that an increased release did not increase intraoperative blood loss (500 ml vs 600 ml), which corresponds with our clinical impression, that cutting through the lamina with an ultrasonic bone scalpel result in very little additional bleeding. This is in contrast to more extensive releases like Ponte osteotomies, which have been associated with greater blood loss and complication rates [29]. The preservation of the superior facet in this technique likely contributes to its safety profile while still achieving the desired flexibility for an opening osteotomy [30].

### Limitations and perspectives

This was a retrospective longitudinal design studying two consecutive time periods which introduces biases in terms of surgical technique and overall surgical strategy. Kyphosis restoration can also be achieved by more bending of the convex rod which may come at the expense of less derotation. Kyphosis restoration in AIS has gained increased attention in recent years and higher surgeon awareness on this element of correction could bias the results. Our research group has published on the issue of kyphosis since 2016; hence, this has been a longstanding focus at our center.

Emerging radiographic parameters may serve as valuable surgical guides. For example, the T4-L1-hip axis has shown potential as an alignment target in adult spinal deformity. Further research is needed to determine whether these parameters could also benefit patients with AIS.

We did not use biplanar X-rays and therefore cannot account for the postoperative derotation. While the results are promising, the relatively short follow-up and the focus on radiographic outcomes are limitations. Future prospective studies should include patient-reported outcomes and longer follow-up periods to assess the clinical impact of improved kyphosis restoration. In addition, biomechanical studies could elucidate the exact mechanism by which the posterior release facilitates kyphosis restoration. The results support the use of wide posterior osseo-ligamentous release in hypokyphotic AIS patients. This technique offers a balanced approach to achieving three-dimensional correction, addressing the often-neglected sagittal plane without compromising safety or coronal correction. The ability to restore normal kyphosis in a higher percentage of patients may lead to improved long-term outcomes and patient satisfaction.

## Conclusion

This study provides compelling evidence for the efficacy of wide posterior osseo-ligamentous release in restoring thoracic kyphosis in AIS patients, particularly in cases of preoperative hypokyphosis. The technique appears to offer a safe and effective method for achieving a balanced three-dimensional correction in AIS surgery. 

## Data Availability

The data that support the findings of this study are available from the corresponding author, [S.O.], upon reasonable request.
